# The “right” side of sleeping: laterality in resting behaviour of Aldabra giant tortoises (*Aldabrachelys gigantea)*

**DOI:** 10.1007/s10071-021-01542-z

**Published:** 2021-08-09

**Authors:** Caterina Spiezio, Camillo Sandri, Flavien Joubert, Marie-May Muzungaile, Selby Remy, Paola Mattarelli, Barbara Regaiolli

**Affiliations:** 1 Parco Natura Viva–Garda Zoological Park, Bussolengo, Italy; 2 Department of Agricultural and Food Sciences, University of Bologna, Bologna, Italy; 3 Ministry of Agriculture, Climate Change and Environment, Victoria, Republic of Seychelles; 4 Biodiversity Conservation and Management Division, Ministry of Agricolture, Climate Change and Environment, Victoria, Republic of Seychelles; 5 Seychelles National Parks Authority, Victoria, Republic of Seychelles

**Keywords:** Giant tortoises, Sleep-like behaviour, Behavioural asymmetries, Seychelles Islands

## Abstract

Although some studies investigated lateralization in reptiles, little research has been done on chelonians, focusing only on few behaviours such as righting response and escape preference. The aim of this study was to investigate lateralization in Aldabra giant tortoises (*Aldabrachelys gigantea*), focusing on asymmetrical positioning of the limbs and the head during resting behaviour, called sleep-like behaviour, involving both wild tortoises and individuals under human care. Subjects of the study were 67 adult Aldabra tortoises (54 free ranging on Curieuse, 13 under human care in Mahè Botanical Garden). For each tortoise observed during sleep-like behaviour, we recorded the position of the head (on the left, on the right or in line with the body midline) and we collected which forelimb and hindlimb were kept forward. Moreover, the number of subjects in which limbs were in a symmetrical position during the sleep-like behaviour was recorded. Based on our results, the number of tortoises with asymmetrical position of head and limb was higher (head: 63%; forelimbs: 88%; hindlimbs: 70%) than the number of tortoises with symmetrical position of the head and the limb. Regarding the head, throughout the subjects found with the asymmetrical position of the head during sleep-like behaviour, tortoises positioning the head on the right (42%) were more than those sleeping with the head on the left (21%). We found a relationship between the position of the forelimbs and hindlimbs during sleep-like behaviour. We reported no differences between Mahè (under human care) and Curieuse (wild) tortoises. Findings of this preliminary study underlined traces of group-level lateralization in head positioning during the sleep-like behaviour, possibly due to a left-eye/right-hemisphere involvement in anti-predatory responses and threatening stimuli as reported in reptiles and other vertebrates. This study aims at adding data on brain lateralization, often linked to lateralized behaviours, in reptiles, especially in chelonians.

## Introduction

Lateralization has been reported in several taxa, ranging from invertebrates (Rogers and Vallortigara 2008; Frasnelli et al. 2012; Versace and Vallortigara 2015) to vertebrates (Vallortigara et al. 2011; Rogers and Vallortigara 2015). Lateralized responses are widespread in different ecological and behavioural settings and they have been commonly described in feeding, predatory, and exploratory contexts involving asymmetrical activation of the brain (Cozzutti and Vallortigara 2001; Palleroni and Hauser 2003; Vallortigara and Rogers 2005; Chivers et al. 2016; Kurvers et al. 2017). Among reptiles, different studies considered behavioural lateralization, focusing especially on anti-predatory and escape responses (Bisazza et al. 1998; Stancher et al. 2018). In the Iberian wall lizard (*Podarcis hispanica*) and in geckos (*Tarentola angustimentalis*) the presence of a right preference for refuge use suggested a left-eye/right-hemisphere advantage for monitoring threats (Garcìa-Muñoz et al. 2012; 2013), that has been reported also in the common wall lizard (*Podarcis muralis*) (Bonati et al. 2008, 2010; Martin et al. 2010; Csermely et al. 2011; Bonati et al. 2012). This left eye/right hemisphere involvement in predatory and anti-predatory behaviour as well as in the control of threatening stimuli has been commonly described in different species of fishes, toads and birds (Bisazza et al. 1998; Vallortigara et al. 1999; Lippolis et al. 2002; Vallortigara and Rogers 2005; Koboroff et al. 2008; Stancher et al. 2018). Regarding lateralization in reptile inactive behaviour, that has been investigated in the current study, past research on snakes revealed an individual-level preference of some individuals for coiling clockwise or anti-clockwise spirals during hibernation and resting (Roth 2003; Heatwole et al. 2007). Although several taxa have been involved in laterality studies, little research has been done on chelonians, a very ancient group of vertebrates (Cleary et al. 2020), focusing only on few behaviours such as righting response (returning upright after being overturned on the carapace) (Stancher et al. 2006; Malashichev 2016; Smith et al. 2017) and escape preference (Pellitteri-Rosa and Gazzola 2018). In particular, European pond turtles (*Emys orbicularis*) have been found to show a left-side preference for escape and righting responses (Pellitteri-Rosa and Gazzola 2018), whereas no group-level bias for righting was found in green turtles (*Chelonia mydas*) and olive Ridley turtles (*Lepidochelys olivacea*) (Malashichev 2016), painted turtles (*Chrysemys picta*) and Eastern musk turtles (*Sternotherus odoratus*) (Smith et al. 2017). Moreover, leatherback turtles (*Dermochelys coriacea*) have been found to show a population-level right bias in flipper use during nesting (Sieg et al. 2010), underlining the possible presence of other lateralized behaviours in chelonians. Regarding terrestrial species, a right bias has been reported in Hermann’s tortoises (*Testudo hermanni*) for righting (Stancher et al. 2006) and for starting to move from a resting position (Sovrano et al. 2017). More research is needed to investigate the lateralization of chelonians, focusing on a greater number of species, especially land tortoises.

Desert tortoises (*Gopherus agassizii*) extend their limbs and head in the warmest hours of the day and during periods of basking, when the body temperature increases rapidly. This posture would allow tortoises to offload heat and has been described behaviour as a thermoregulatory behaviour (Voigt 1975). Similarly, in Galapagos tortoises (*Geochelone elephantopus*), during the day most tortoises sleep in the open with the head and limbs extended (sleep-like behaviour), whereas during evening/night hours sleeping occurs with head and limbs withdrawn into the carapace (Hayes et al. 1992). As no native predators are reported for the Galapagos tortoises on Galapagos, the postures of the sleep-like behaviour during the day may imply a thermoregulatory rather than an antipredatory function (Hayes et al. 1992). A similar posture during daytime resting has been observed also in the Seychelles giant tortoises involved in the current study that focused specifically on the laterality of this posture, hereafter referred to as sleep-like behaviour.

The Aldabra giant tortoises (*Aldabrachelys gigantea*) of the Seychelles islands were the subjects of this research. The Aldabra giant tortoise is the only extant species of giant tortoises in the Indian Ocean, and it is a threatened species listed as Vulnerable to extinction according to the IUCN Red List^™^ (Tortoise and Freshwater Turtle Specialist Group 1996; Balmer et al. 2011). The aim of this study was to investigate lateralization in Aldabra giant tortoises by focusing on asymmetrical positioning of the head and the limbs during sleep-like behaviour. We hypothesized that as described for the Galapagos giant tortoises, also the Seychelles giant tortoises would extend the limbs in the sleep-like posture for thermoregulation during the day (Hayes et al. 1992). Due to the size and natural lack of mammalian predators, adult Aldabra giant tortoises were thought to have no predators in the wild, although humans can be considered as predators of this species. The sleep-like behaviour can be considered a thermoregulatory posture, it could involve a trade-off with predation risk and can be characterized by functional biases in limb position. Indeed, keeping the right or left forelimb and/or hindlimb extended forward might be determinant when standing or when starting locomotion (Tommasi and Vallortigara 1999; Stor et al. 2019). In addition, researchers have assumed that predation risk is increased during sleeping and most animals seek safe sleeping sites or sleep in groups to obtain communal protection. Those large herbivores that cannot find safe sleeping sites appear to have smaller amounts of sleep and sleep less deeply (Siegel 2015). This could be also the case of Aldabra giant tortoises, a giant herbivore reptile, in which the lateralization during the less deeply sleep-like behaviour during the day may also help in controlling the surrounding environment and respond to threatening situations.

To our knowledge, this is the first study on lateralization in Aldabra giant tortoises, an ancient vertebrate species, living in their native area, the Seychelles Islands. The goal of this study was to further document behavioural lateralization in reptile species, focusing on the sleep-like behaviour of Aldabra giant tortoises. Also, we investigated functions of the asymmetry of this behaviour to find a possible link with cerebral lateralization. This study aims at adding data on brain lateralization, often linked to lateralized behaviours, in reptiles, especially in chelonians.

## Materials and methods

### Study subjects and area

This study was carried out with 67 Aldabra giant tortoises living in the Seychelles Islands, 54 wild tortoises living on Curieuse Island and 13 tortoises kept under human care on Mahè Island. All tortoises were adults of unknown sex. Tortoises were identified through letter and number combination on the shell or through physical characteristics of the scutes. Tortoises on Curieuse were free ranging and had access to the native vegetation of the island, particularly grass, leaves, fruits, and flowers. Ten tortoises on Mahè were housed in the Botanical Garden (BG), whereas three subjects were kept in the Coral Strand Hotel (CSH). Tortoises in the BG were housed in a 1000 m^2^ enclosure on different levels, containing rocks, sandy area, water and muddy pools and they were fed with a main meal of local browse, although part of the food amount (banana leaves) was provided by visitors during the day. Occasionally, tortoises were also provided with fruits. Water was available ad libitum in a pool. CSH tortoises were housed in a 70 m^2^ grassy enclosure containing a sandy area and a water pool. They were fed with fresh leaves and vegetables once a day.

The study was carried out through the behavioural observation of the tortoises during their normal daily activity, using non-invasive techniques. There was no intervention upon tortoises and no interaction with them as data were collected being at a minimum distance of two meters from individuals. The study was done with the permission of the Biodiversity Conservation and Management Division of the Ministry of Environment, Energy and Climate Change of Seychelles, under the “Partnership Agreement” between Parco Natura Viva and the Seychelles National Parks Authority (SNPA).

### Data collection

**Fig. 1:**
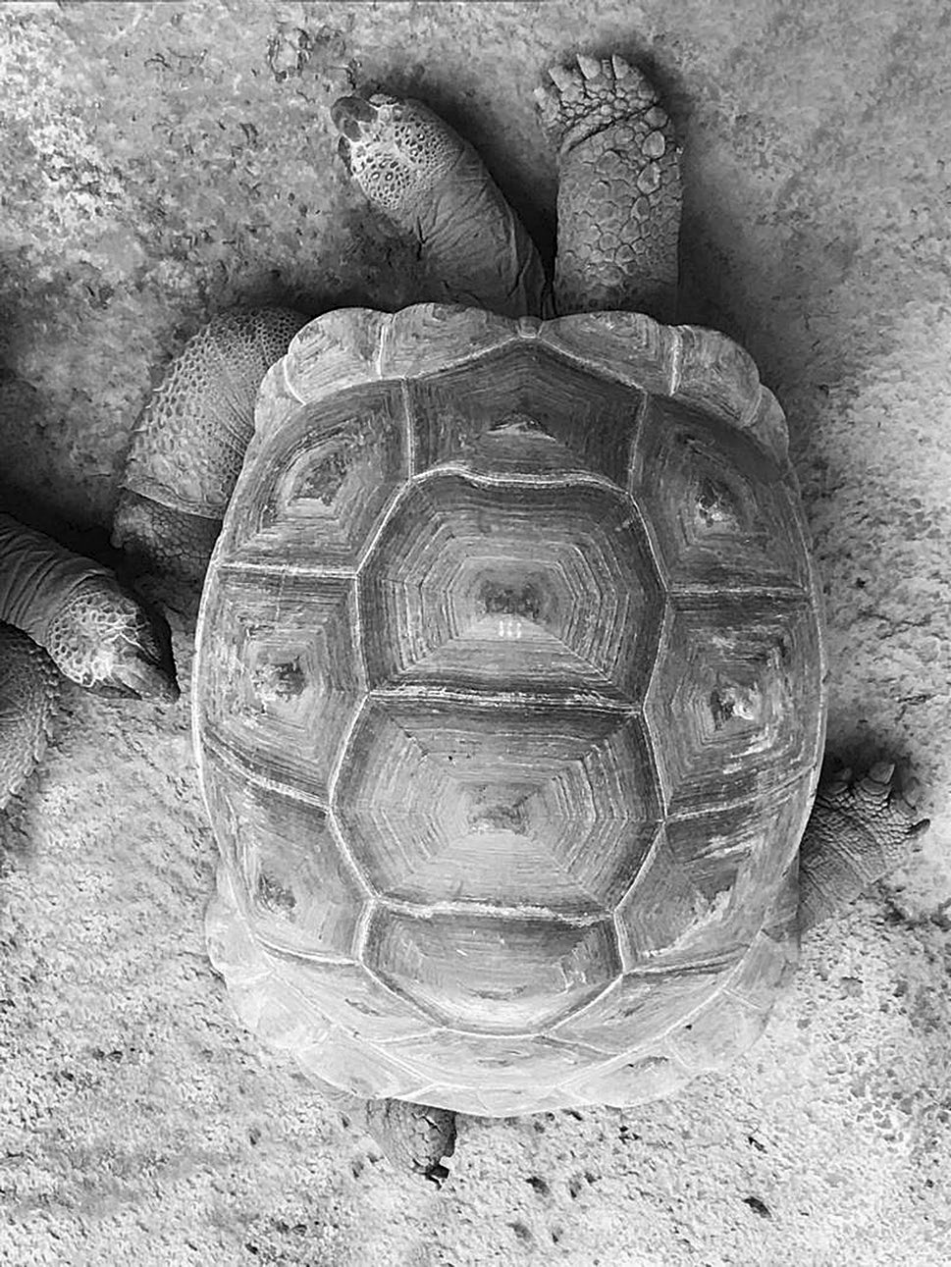
Aldabra giant tortoise in sleep-like behaviour. In this picture, the tortoise is in sleep-like behaviour with the right forelimb and hindlimb extended forward, whereas the left forelimb and hindlimb are extended backward. The head is on the left of the body midline

**Fig. 2:**
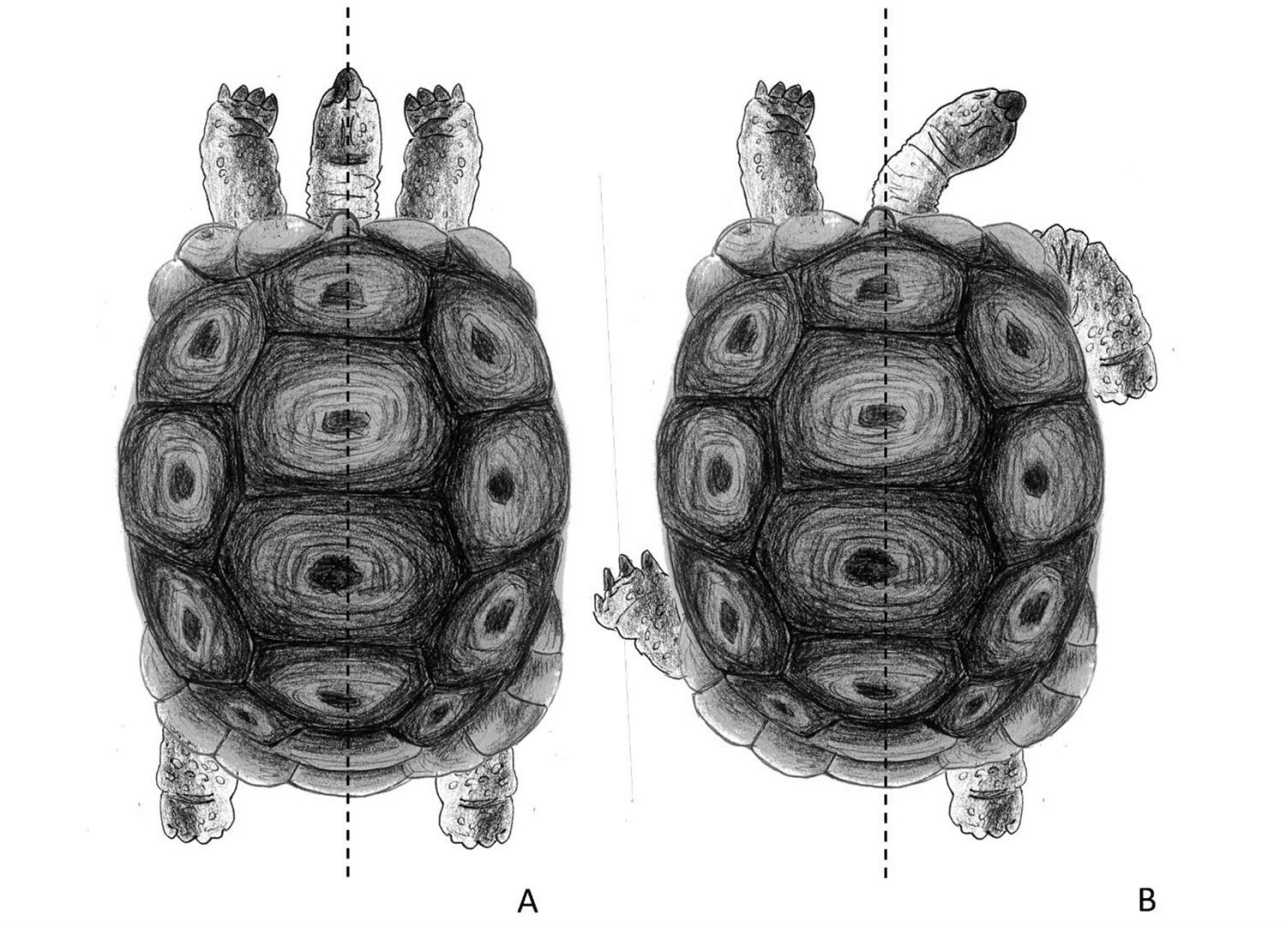
Aldabra giant tortoise in sleep-like behaviour. In example A, the head is in line with the body midline, both forelimbs are extended forward and both hindlimbs are extended backward (symmetrical). In example B, the head is on the right, the left forelimb and hindlimb are extended forward, whereas the right forelimb and hindlimb are bent backward

The study was carried out sampling all tortoises during sleep-like behaviour on both Mahè and Curieuse and data were collected in February 2020. For each tortoise encountered that was performing this behaviour, side preferences in the position of the head, forelimbs and hindlimbs were collected. We chose locations that allowed us to optimize data collection, such as spots where several tortoises were present. We only sampled each spot/area once, to avoid the risk of recording several times the same tortoise. During the day, tortoises were considered in sleep-like behaviour if the neck was laying on the ground and the eyes were closed. In particular, Aldabra tortoises sleep in the open with head and limbs extended, sprawled on the ground (Swingland 1989) (Fig. 1). Based on our field observation, the head/neck can be kept either in line with the body longitudinal midline or on one side (to the left or to the right of the body axis), sometimes leaning on one limb. The forelimbs can be kept both forward and both backward, symmetrically to the body midline. However, commonly one forelimb is forward, whereas the other one is backward. In particular, the forelimb that is kept forward has the palm face of the foot on the ground and it may be the first one that is used when starting to move. The forelimb bent backward is relaxed with the dorsal face on the ground (Figs. 1, 2). Similar positioning applies to the hindlimbs that can be both extended backward and parallel, bent forward or alternated (one forward and one backward) (Fig. 2). The hindlimb forward has the palm of the foot on the ground, whereas the other one is relaxed with the dorsal face on the ground. In the current study, we focused on sleeping tortoises and for each subject, we recorded whether the head was kept in the midline or on the right/left side of the body midline. The head was considered in an asymmetrical position when the neck (and the head) was laying on the ground, bent on a horizontal plane to one side of the tortoise midline (the line passing through the nuchal or cervical scute and the supracaudal scutes) (Fig. 2). We did not collect data on tortoises with an ambiguous position of the neck/head (e.g., neck slightly bent). Regarding the forelimbs and hindlimbs, for each tortoise, we collected which limb (right or left) was extended forward during sleeping or whether both limbs had the same orientation and were in a symmetrical position (both limbs extended forward or backward). We used “ad libitum” sampling to record all occurrences of sleep-like behaviours (Altman 1974; Chapelain et al. 2015) and we collect data by direct observation. We aimed at investigating population-level laterality for sleep-like behaviour (single events of sleep-like posture) and test whether the behaviour varies between populations (wild vs. under human care). Thus, we avoided collecting repeated measures for a tortoise and each subject was sampled once over the study period, to be sure that data were strictly independent of each other (Chapelain et al. 2015). In other words, the dataset was made of the number of subjects sleeping with different orientation of the limbs and heads.

### Data analysis

Binomial tests were used to compare the number of lateralized and non-lateralized tortoises in head, forelimb and hindlimb positioning on the total number of subjects observed. The same test was used within lateralized tortoises to test whether subjects with right or left biases in the position of the head, forelimbs and hindlimbs were equally distributed (García-Muñoz et al. 2012). We also calculated a Handedness Index (HI) to quantify laterality on a continuum in the whole sample and in each location using the formula: HI = (N right-biased tortoises − N left-biased tortoises)/(N right-biased tortoises + N left-biased tortoises). The HI was calculated for the position of the head, forelimbs and hindlimbs, with negative values indicating a bias toward the left and positive values indicating a bias toward the right (Hopkins 1999; Chapelain et al. 2015). In addition, a Laterality Score was assigned to each tortoise based on the position of the head and limbs during sleeping. For the head, the Laterality Scores were 1—head on the left, 2—head in line with the body midline, 3—head on the right. For the forelimbs, the Laterality Scores were 1—forelimb extended forward on the left, 2—both forelimbs extended forward, 3—forelimb extended forward on the right. For the hindlimbs, the Laterality Scores were 1—hindlimb extended forward on the left, 2—both hindlimbs extended backward, 3—hindlimb extended forward on the right. As Shapiro–Wilk Goodness-of-Fit tests revealed that the Laterality Scores were not normally distributed, statistical analyses were done using non-parametric statistic tests. To investigate the relationships between the position of the forelimbs and the hindlimbs as well as between the position of the limbs and the head, Spearman correlations were done between the Laterality Scores calculated for head, forelimbs and hindlimbs. Bonferroni adjustment was adopted to correct the *p* value for multiple comparisons (corrected *p* value = 0.05/3 = 0.017). Finally, the Laterality Scores were compared between locations (Mahè and Curieuse) using a Mann–Whitney test.

## Results

### Lateralization in head, forelimb and hindlimb positioning

**Fig. 3:**
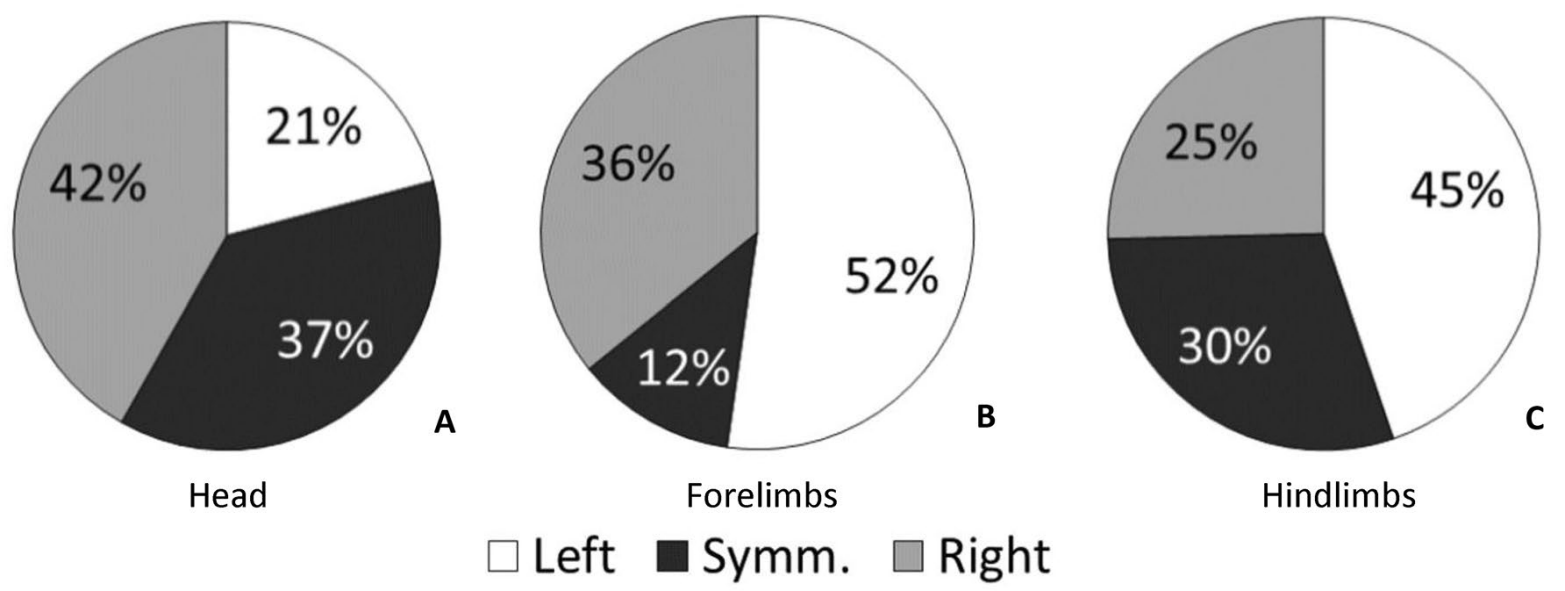
Number of tortoises (%) with different sleeping position of the head (**a**), forelimbs (**b**), and hindlimbs (**c**). The pie charts show the % of tortoises sleeping with the head on the left or on the right, and with the right and the left limbs extended forward. Dark grey slices indicate % tortoises with head and limbs in a symmetrical position (Symm)

First, for the head positioning, 63% of the tortoises were lateralized. Among lateralized subjects, 21% of the tortoises were in a sleep-like behaviour with the head on the left, 42% on the right (HI: 0.333). The remaining 37% of the tortoises were in a sleep-like behaviour with the head in line with the body midline (Fig. 3a, Table 1). Binomial test revealed a slightly significant difference in the number of lateralized and non-lateralized tortoises (*p* = 0.049). Within lateralized tortoises, the number of subjects in a sleep-like behaviour with the head on the right was significantly higher than the number of those with the head on the left (*p* = 0.044).

**Table 1:** Number of tortoises with different positions of the head, forelimbs and hindlimbs during sleep-like behaviour

		Mahè	Curieuse
Head			
Left		5	9
Midline		1	24
Right		7	21
LS		3 (2)	2 (1)
HI		0.167	0.400
Forelimbs			
Left		8	27
Symm		3	5
Right		2	22
LS		1 (1)	1.5 (2)
HI		− 0.600	− 0.102
Hindlimbs			
Left		8	22
Symm		5	15
Right		0	17
LS		1 (1)	2 (2)
HI		− 1	− 0.128

For different body parts and for different locations (Mahè, under human care and Curieuse, free ranging), the table reports the number of subjects in which the head was on the left, in line with the body axis (Midline) or on the right. For forelimbs and hindlimbs, the table reports the number of tortoises sleeping with the right or left limb extended forward or with both limbs in a symmetrical position (Symm.). The last rows report the median (interquartile range) of the Laterality Score (1: Left, 2: Symm.; 3: Right) and the HI calculated for each location

In the case of the forelimbs, 88% of the tortoises were lateralized. Among lateralized subjects, 52% of the tortoises were in a sleep-like behaviour with the left forelimb forward, 36% with the right forelimb forward (HI: -0.186). The remaining 12% of the tortoises were in a sleep-like behaviour with both limbs on the same side (Fig. 3b, Table 1). Binomial test revealed a highly significant difference in the number of lateralized and non-lateralized tortoises (*p* < 0.001). Within lateralized tortoises, no significant difference was found between the number of tortoises sleeping with the right and the left forelimb forward (*p* = 0.193).

In the case of the hindlimbs, 70% of the tortoises were lateralized. Among lateralized subjects, 45% of the tortoises were in a sleep-like behaviour with the left hindlimb extended forward, 25% with the right hindlimb extended forward (HI:  − 0.277). The remaining 30% of the tortoises were in a sleep-like behaviour with both limbs extended forward symmetrically (Fig. 3c, Table 1). Binomial test revealed a significant difference in the number of lateralized and non-lateralized tortoises (*p* = 0.001). Within lateralized tortoises, no significant difference was found between the number of tortoises in a sleep-like behaviour with the right and the left hindlimb extended forward (*p* = 0.079).

### Relationship between head, forelimb and hindlimb positioning

Then, we verified whether the position of the head could be related to that of the limbs by correlating with each other the Laterality Scores calculated for different body parts. First, a strong significant positive correlation was found between the forelimbs and the hindlimbs when considering the Laterality Scores (*rho* = 0.725, *p* = 0) (Bonferroni-adjusted *p* value: 0.017), suggesting similar side biases in the forelimbs and hindlimbs. Namely, when the left forelimb is extended forward, also the left hindlimb is likely to be extended forward. On the other hand, no correlation was found between the position of the head and that of the forelimbs (*rho* =  − 0.201, *p* = 0.104) (Bonferroni-adjusted *p* value: 0.017). The same result emerged considering the head and the hindlimbs (*rho* =  − 0.091, *p* = 0.464) (Bonferroni-adjusted *p* value: 0.017).

We assessed intra-individual consistency in head and limb posture, to examine whether the individuals keep their head bent and limbs extended forward on the same side. We first assessed the consistency of all tortoises found in sleep-like behaviour with the head bent, and both limbs extended forward on the same side (e.g., head on the right and right forelimb and hindlimb extended forward). We found that 29% of individuals observed during sleep-like behaviour was consistent with the head bent and the limbs extended forward on the same side (25% of Mahè tortoises and 30% of Curieuse tortoises). These findings show that approximately 1/3 of the study population keep the head bent on the same side of both forelimb and hindlimb. We also examined the consistency within individuals of the position of the forelimbs and the hindlimbs: 60% of tortoises on Mahè were found with forelimbs and hindlimbs extended forward on the same side (or both parallel), whereas the 70% of the tortoises on Curieuse were found with forelimbs and hindlimbs extended forward on the same side (or both parallel).

### Mahè vs Curieuse

**Fig. 4:**
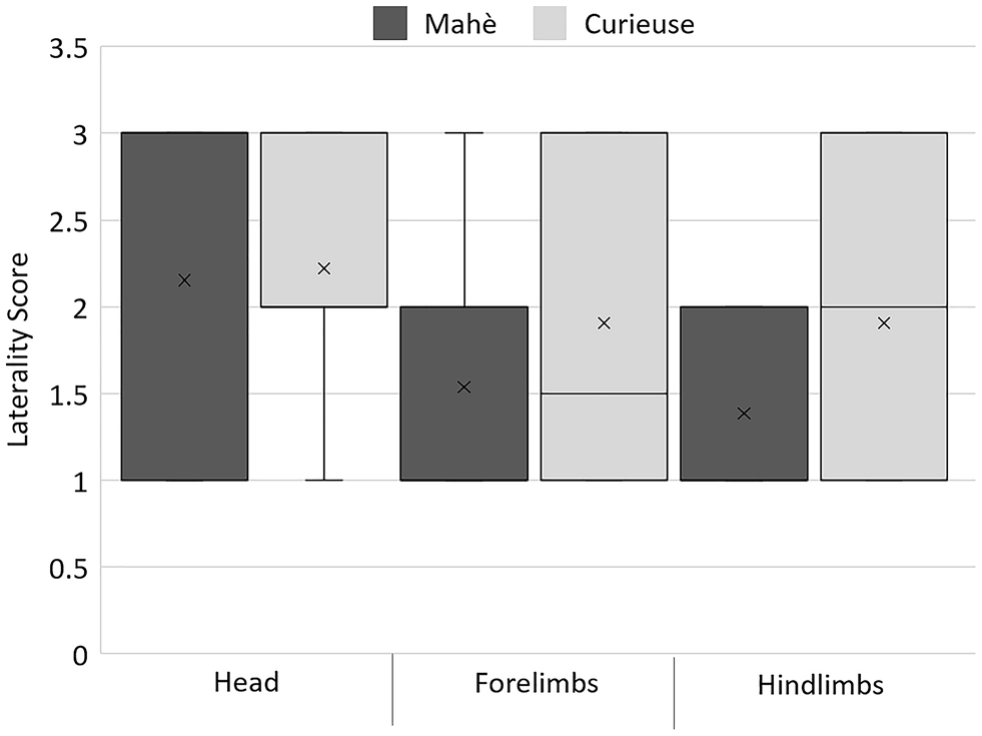
Laterality Score of sleeping Aldabra tortoises on Mahè (under human care) and Curieuse (free ranging). Left-biased tortoises for the position of the head, or the limbs were assigned with a score of 1, tortoises with asymmetrical positions of the head or the limbs had a score of 2, right-biased tortoises for the position of the head or the limbs had a score of 3. The horizontal lines within the box indicate the medians, crosses indicate means, boundaries of the box indicate the first and third quartile. The whiskers extend up from the top of the box to the largest data element that is less than or equal to 1.5 times the interquartile range (IQR) and down from the bottom of the box to the smallest data element that is larger than 1.5 times the IQR. Values outside this range are considered outliers and are drawn as points

Finally, we investigated whether patterns of lateralization differed between Mahè (under human care) and Curieuse (free ranging) tortoises by comparing the Laterality Score between locations. No significant differences between Mahè and Curieuse tortoises were found considering the position of the head (*U* = 348, *p* = 0.968), forelimbs (*U* = 282.5, *p* = 0.280) and hindlimbs (*U* = 235.5, *p* = 0.069) (Table 1, Fig. 4). Considering group-level lateralized tortoises, the HI for the head was 0.167 for Mahè and 0.400 for Curieuse; the HI for the forelimbs was  − 0.600 for Mahè and  − 0.102 for Curieuse; the HI for the hindlimbs was  − 1 for Mahè and  − 0.128 for Curieuse.

## Discussion

The main findings of this study on Aldabra giant tortoises are (1) a significantly higher number of lateralized than non-lateralized individuals in positioning of the head, forelimbs and hindlimbs during sleep-like behaviour; (2) a significant preference for performing a sleep-like behaviour with the head on the right of the body midline; (3) coordination in the position of the forelimbs and hindlimbs on the same side of the body.

In the current study, the Aldabra giant tortoises observed during the day were found sleeping in the sleep-like posture described by Hayes and colleagues (1992) for the Galapagos giant tortoises, with forelimbs and hindlimbs sprawled on the ground (Swingland 1989), extended forward or backward. More than 60% of the study tortoises were found to sleep with the head, the forelimbs and/or the hindlimbs in an asymmetrical position, especially regarding the limbs (more than 80%). Indeed, the number of the tortoises observed during the sleep-like behaviour with lateral biases in head positioning was significantly more than tortoises found sleeping with the head in line with the body midline. Similarly, tortoises observed with lateral biases in forelimb and hindlimb positioning during the sleep-like posture were significantly more than those found sleeping with both front and back limbs in the same direction and a strong group-level significant difference was found (*p* ≤ 0.001). These findings are consistent with previous literature in different vertebrate taxa, suggesting that being lateralized might be advantageous and, therefore, more likely than showing no lateralization (e.g., Rogers 2002; Rogers et al. 2004; Magat and Brown 2009; Regaiolli et al. 2021), even during inactive behaviours such as sleeping (Roth 2003; Heatwole et al. 2007).

Throughout the subjects observed sleeping with asymmetric sleep-like posture, despite the inter-individual variation of side in forelimb and hindlimb positioning, tortoises found in a sleep-like behaviour with the head on the right were significantly more common (42%) than those with the head on the left (21%). A plausible explanation could be that when the head is resting on the right, tortoises have the left eye available to monitor the surrounding in the case of an alarming or threatening situation or stimulus. Previous studies on reptiles reported a similar right bias in refuge selection in lizards, specifically *Podarcis hispanica* and *Tarentola angustimentalis*, underlining a left-eye preference for monitoring threatening stimuli (García-Muñoz et al. 2012, 2013) and in *P. muralis*, showing a left-eye involvement in the mediation of predatory inputs (Martìn et al. 2010; Bonati et al. 2008, 2010, 2012). In addition, the right hemisphere seems to control rapid responses to changes in the surrounding environment (Andrew and Rogers 2002). Similarly, a recent study on freshwater turtles reported that European pond turtles showed a lateralization of anti-predatory behaviour, escaping significantly more on the left side in the presence of a predatory threat (Pellitteri-Rosa and Gazzola 2018). Lateralization in eye use seems widespread, particularly in ectotherms, in which the right eye/left hemisphere is involved in predatory behaviour and food searching whereas the left eye/right hemisphere seems to control predator monitoring (Bonati et al. 2010). Even though sleep can be seen as an adaptive state, benefiting animals by increasing the efficiency of their activity, the sleep also allows, in some circumstances, rapid arousal for dealing with threatening situations, and responding to environmental changes. Indeed, many organisms can reduce sleep for long periods of time in which a selective advantage can be obtained by continuous waking (Siegel 2008, 2015) and this may be the case of the sleep-like behaviour observed during the day in land tortoises: individuals are resting but ready to react in the presence of a threat.

Based on our results, we reported a strong positive correlation between the Laterality Score of the forelimbs and the hindlimbs in the study Aldabra giant tortoises. Similarly, especially regarding the limbs, 60 to 70% of the tortoises was found with the forelimb and hindlimb on the same side of the body extended forward. Namely, if the right forelimb is extended forward during sleep-like behaviour, also the right hindlimb is extended forward (Fig. 1). Possibly, this posture might be advantageous at the end of the sleeping period, as having the limbs on one side extended in the same direction might facilitate the start of locomotion. Having limbs and head extended seems to be a thermoregulation need (Voigt, 1975; Hayes et al. 1992). However, the laterization of the extended limb and the position of the head may have other adaptive functions. In particular, postural asymmetries in Aldabra giant tortoises may have the same function of lateralization described in other reptiles (García-Muñoz et al. 2012, 2013; Martìn et al. 2010; Bonati et al. 2010; 2012; Pellitteri-Rosa and Gazzola 2018) and in vertebrate species in general (Vallortigara et al. 1999; Lippolis et al. 2002; Vallortigara and Rogers 2005; Koboroff et al. 2008) suggesting a left-hemisphere advantage in processing anti-predatory responses and threatening situations.

This study represents a preliminary investigation of group-level lateralization in Aldabra giant tortoises and at the moment it seems to be the first attempt to study lateralization in wild individuals of this threatened species on Curieuse Island. Our results suggest the presence of traces of lateralization in wild Aldabra giant tortoise population, especially regarding the position of the head, deserving future research. In addition, these findings might be explained by an involvement of the right hemisphere in predatory responses and threatening situations that has been described in other reptiles, specifically in chelonians, as well as in other vertebrates (Bisazza et al. 1998; Vallortigara et al. 1999; Lippolis et al. 2002; Vallortigara and Rogers 2005; Koboroff et al. 2008; Stancher et al. 2018).

In conclusion, the population of Aldabra giant tortoises of the current study showed asymmetry in the position of forelimbs and hindlimbs during sleep-like behaviour, although we found no group-level biases considering the limbs. On the other hand, the position of the head was significantly skewed, as most of the observed tortoises were found during the sleep-like behaviour with the head on one side, in particular the right, allowing the left eye to monitor the surrounding environment and control possible threats. As the Chelonian is an ancient group of vertebrates and lateralized behaviours have been linked to hemispheric specialization, the evidence of lateralized behaviours at group level in these ancient reptiles may provide data on the evolutionary origins of vertebrate brain lateralization. Moreover, these findings seem to support the advantageous nature of the behavioural asymmetry linked to the cerebral lateralization in the everyday life and the importance of the environment and habits in the emergence and evolution of lateralization.

It might be interesting to replicate the study in a far bigger pool of tortoises on other islands of Seychelles. Moreover, we are planning future studies to collect repeated measures of sleep-like position as well as starting locomotion in Aldabra giant tortoises, to investigate individual-level preferences in forelimb and hindlimb positioning and to assess possible advantages and function of asymmetries in the sleep-like behaviour reported in the current study.
